# Editorial: Chemicals in the Environment and Brain Development: Importance of Neuroendocrinological Approaches

**DOI:** 10.3389/fnins.2017.00133

**Published:** 2017-03-21

**Authors:** Fumihiko Maekawa, Kazuaki Nakamura, Shoji F. Nakayama

**Affiliations:** ^1^Center for Health and Environmental Risk Research, National Institute for Environmental StudiesTsukuba, Japan; ^2^Division of Life Science, Graduate School of Science and Engineering, Saitama UniversitySaitama, Japan; ^3^Department of Pharmacology, National Research Institute for Child Health and DevelopmentSetagaya, Japan

**Keywords:** children's health, developmental disorder, environment, chemicals, brain

In the past three decades, a sharp increase in the number of children diagnosed with neurodevelopmental disorders has been observed; the reason for this is not well-explained (Weintraub, 2011). The human genome does not change rapidly; this suggests that non-genetic factors are the driving forces of this dramatic surge. Several reports, including epidemiological studies, have found an association between *in utero* and childhood exposure to certain environmental chemicals and children's brain development. Yet, the mechanisms by which these chemicals impair brain development and function are not fully understood. In addition, how these chemicals enter and accumulate in the brain are still unknown. Experimental approaches are essential to understand how those harmful chemicals enter children's brain and pose discrete effects on specific brain sites. These approaches include the following: improvement of technologies for the detection and measurement of neuroendocrinological and behavioral changes in animal models: development of analytical methods for the identification and quantification of chemicals and their metabolites in the brain; development of *in vitro* cell line assays; and imaging technologies to illustrate cellular functions.

In this Research Topic, we collected articles that provide state-of-the-art science and technologies that can help us identify environmental chemicals that influence brain development. We also included articles that lead to a better understanding of the actions and dynamics of these chemicals. As summarized in the review by Fujiwara et al. certain chemical exposures such as atmospherically released chemicals (volatile organic chemicals and pesticides), metals, endocrine disruptors, and psychoactive pharmaceuticals are associated with an increased risk of autism spectrum disorder, a neurodevelopmental disorder. Thus, we especially encouraged researchers to submit their works that are related to fetal and early childhood (i.e., early-life) exposure to these chemicals.

Among volatile organic chemicals, Win-Shwe et al. revealed that early-life exposure to secondary organic aerosol, a component of particulate matter (PM), especially PM_2.5_, impairs social memory in adulthood; this was demonstrated using the murine three-chamber test. As for pesticides, recent studies have suggested the possibility that neonicotinoids, which are known to overstimulate insect nicotinic acetylcholine receptors and kill insects, also impair neuronal transmission in the mammalian brain. Sano et al. elucidated the effect of developmental exposure to acetamiprid, a neonicotinoid, on murine behavioral profiles in adulthood; their study confirmed the transfer of acetamiprid to the developing brain. They revealed that exposure to acetamiprid induces abnormalities in socio-sexual and anxiety-related behaviors in sex- and dose-dependent manners.

Lead, mercury, and arsenic are infamous as neurotoxic chemicals. Early-life exposures to these metals might be related to the increased risk in neurodevelopmental disorders. Since more than 200 million people worldwide have been estimated to be exposed to arsenic from drinking water and food, experimental studies on its effects on the developing brain are required to evaluate whether early-life exposure to arsenic at environmentally relevant doses causes neurodevelopmental disorders. Aung et al. found that mice exposed to arsenic *in utero* displayed an impaired adaptation to repetitive reversal tasks, one of the typical features of autistic spectrum disorder. They also found that the neurite length of neurons in the prelimbic cortex is significantly reduced in the mice exposed to arsenic *in utero*; this suggests the possibility that impaired formation of neural connections in the prelimbic cortex is one of the causes of the observed behavioral abnormality. In the brain, astrocytes could also be affected by arsenic neurotoxicity. Htike et al. developed a method to evaluate the effect of arsenic exposure on the cell cycle of primary cultured cortical astrocytes using transgenic mice expressing a fluorescence protein indicator, which enabled the visualization of the cell cycle. Using this method, they found that arsenic exposure led to early entry to mitotic S-phase and subsequently induced cell death. The mechanisms of the transport into the brain and the neurotoxicity of metals are not yet fully understood. Ximenes-da-Silva reviewed the biological route through which metal ions is transported and focused on a metal transporter, aquaporin-4. On the toxicity of metals, Harada et al. suggested that gliotransmitter release from astrocytes needs to be investigated as a new target of metal ions.

Among endocrine-disrupting chemicals, bisphenol A has been demonstrated to possess an estrogenic activity in many experimental models. Cano-Nicolau et al. developed a method to detect Cyp19a1 and Cyp19b1 promoter activities by *in vivo* imaging of transgenic zebrafish expressing the green fluorescent protein (GFP) under the control of either Cyp19a1 or Cyp19b1 promoters. They revealed that bisphenol A and its substitutes have strong estrogenic activities. Apart from exhibiting estrogenic activity, bisphenol A and related compounds also have broad toxicological effects. Ling et al. established a method to visualize the neuronal migration in the cerebral cortex using *in utero* electroporation of a plasmid expressing a fluorescent protein. They demonstrated that exposure to bisphenol A during the late embryonic period in mice disturbed neuronal migration and impaired gene expression of neurotrophic factor receptor tropomyosin receptor kinase B (TrkB). The mode of action for endocrine-disrupting chemicals includes epigenetic effects. Derghal et al. reviewed how exposure to certain endocrine-disrupting chemicals changed the expression of microRNA and thereby caused endocrine diseases and disorders.

Exposure to psychoactive pharmaceuticals could affect brain development. Furukawa et al. revealed that the administration of benzodiazepines to mice during the juvenile period caused irreversible learning and memory deficits; this suggests that an extraordinary amount of care is required for prescription of benzodiazepines to juveniles. Steinberg and Moreira reviewed the risk of treatment of pregnant women diagnosed with acute ischemic stroke with recombinant tissue plasminogen activator, which has been shown to have neuroendocrine effects in vasopressin secretion. Although tissue plasminogen activator has been generally avoided in pregnant women, the authors claimed that the treatment risk must be balanced against the potential of maternal health risk of ischemic stroke.

To elaborate the precise mechanism of the effect of early-life exposure to environmental chemicals and use the knowledge for prevention and intervention, the development of alternative animal models that enable the detection of subtle physiological, anatomical, and functional alterations is an urgent matter. Kawashima et al. reviewed how avian models could be used to evaluate developmental abnormalities of the neurologic and reproductive systems. Animal models could be also used to detect compounds that counteract the harmful effects of environmental factors. Ge et al. established the rat model of subclinical hypothyroidism using partial thyroid electrocauterization and found that resveratrol ameliorated the anxiety- and depression-like behaviors. Goto et al. reviewed the murine depression model for future detection of antidepressant-like effects of chemicals.

The articles in this Research Topic, by applying newly established methods, supplied novel information about harmful endpoints of environmental chemicals such as secondary organic aerosol, neonicotinoid, arsenic, bisphenol A, and psychoactive pharmaceuticals. The reviews demonstrated the typical and novel interactions between environmental chemicals and the developing brain. We believe that these studies would lead to further understanding of neurodevelopmental disorders caused by environmental factors.

## Author contributions

All authors listed directly contributed to the writing and approved the manuscript for publication.

## Funding

This work was partly supported by the National Institute for Environmental Studies (1416AT001).

### Conflict of interest statement

The authors declare that the research was conducted in the absence of any commercial or financial relationships that could be construed as a potential conflict of interest.
